# Reply to Chen: Methodological and analytical considerations in the study of H3-Dendra2 distribution

**DOI:** 10.1073/pnas.2536993123

**Published:** 2026-03-06

**Authors:** Anqi Li, Dong Tong, Benjamin Ohlstein, Zheng Guo

**Affiliations:** ^a^Department of Medical Genetics, School of Basic Medicine, Institute for Brain Research, Tongji Medical College, Huazhong University of Science and Technology, Wuhan 430022, China; ^b^Children’s Research Institute and Department of Pediatrics, University of Texas Southwestern Medical Center, Dallas, TX 75235; ^c^Cell Architecture Research Center, Huazhong University of Science and Technology, Wuhan 430030, China

A recent comment by Chen (1) questions some conclusions of our study regarding histone segregation in *Drosophila* stem cells (2). Our detailed point-by-point response, which includes all experimental controls and data re-analyses, is publicly available (3). Herein, we address the central critiques regarding statistical interpretation, methodology, and technical rigor.

**Fig. 1.**
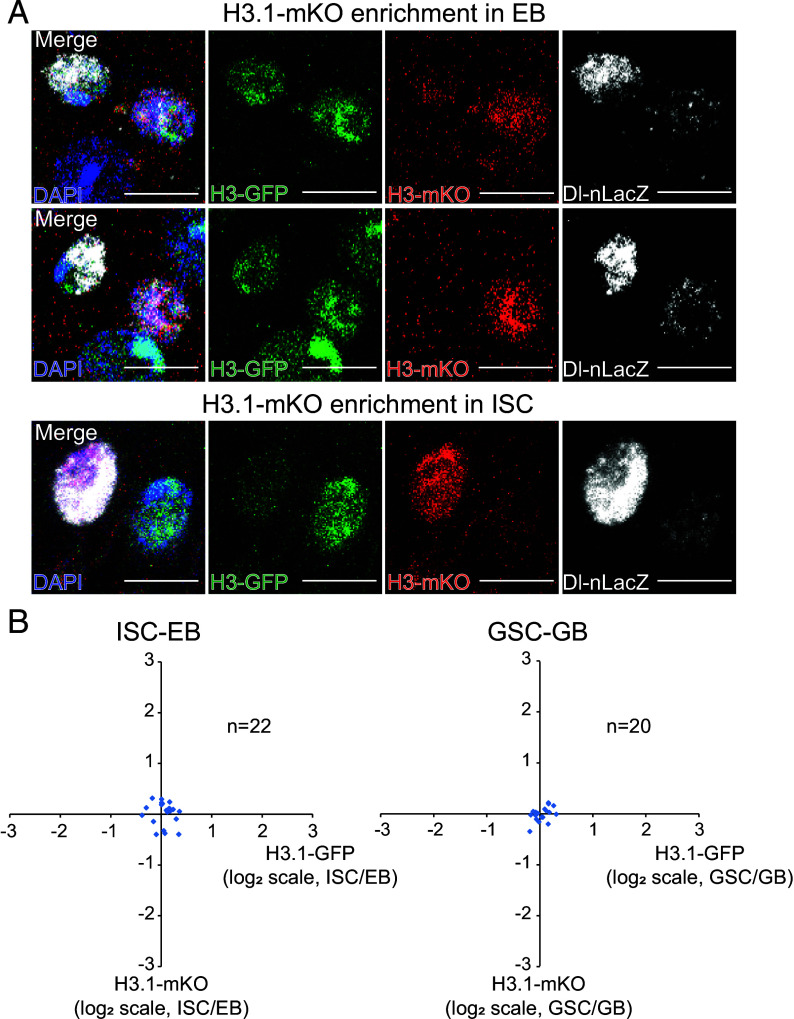
Occasional H3.1-mKO asymmetric distribution in ISC-EB pairs and two-dimensional plot analysis of old and new histone distribution patterns. (*A*) Using the Gal4/UAS Flp-out system, representative images show enrichment of H3.1-mKO (new histone) in the EB or ISC. (*B*) Distribution of H3.1-GFP (old) and H3.1-mKO (new) during ISC asymmetric division (corresponding to ref. 2’s *SI Appendix*, figure S9B) and GSC asymmetric division (corresponding to ref. 2’s *SI Appendix*, figure S10B). [Scale bar: 5 µm (*A*).]

Chen reanalyzed our data obtained using the Gal4/Upstream Activation Sequence (UAS) Flp-out system, correctly noting a statistically significant deviation (*P* < 0.05) from a perfect 1:1 ratio for H3.1 in Intestinal Stem Cell-Enteroblast (ISC-EB) pairs. However, this metric alone cannot support her model of consistent “old” histone (H3.1-Green Fluorescent Protein (GFP)) enrichment in the stem cell (4). A significant *P* value merely rejects the null hypothesis of perfect symmetry; it does not specify the direction of bias. In fact, the mean H3.1-GFP fluorescence ratio (ISC:EB, log_2_ scale) in ISC-EB pairs is less than 0 (*SI Appendix*, figure S8B in ref. 2), which directly contradicts the specific prediction of increased signal in the ISC. Furthermore, our imaging data contradict the predicted unidirectional pattern. We observe H3.1-mKO could be enriched in either the ISC or the EB across different cell pairs (Fig. 1*A*). The variability likely arises because in the constitutive Gal4/UAS Flp-out system, recombination can occur independently in either the ISC or the EB of a pair, not necessarily reflecting a recent mitotic event. To unambiguously assess H3.1-GFP and H3.1-mKO segregation during division, we focused our analysis on mitotic ISC-EB pairs identified by phospho-histone H3 (PH3) staining (*SI Appendix*, figure S9 in ref. 2). The distribution from these dividing cells, visualized in 2D plots (Fig. 1*B*), clusters symmetrically around the origin, supporting a general lack of systematic asymmetry.

**Fig. 2.**
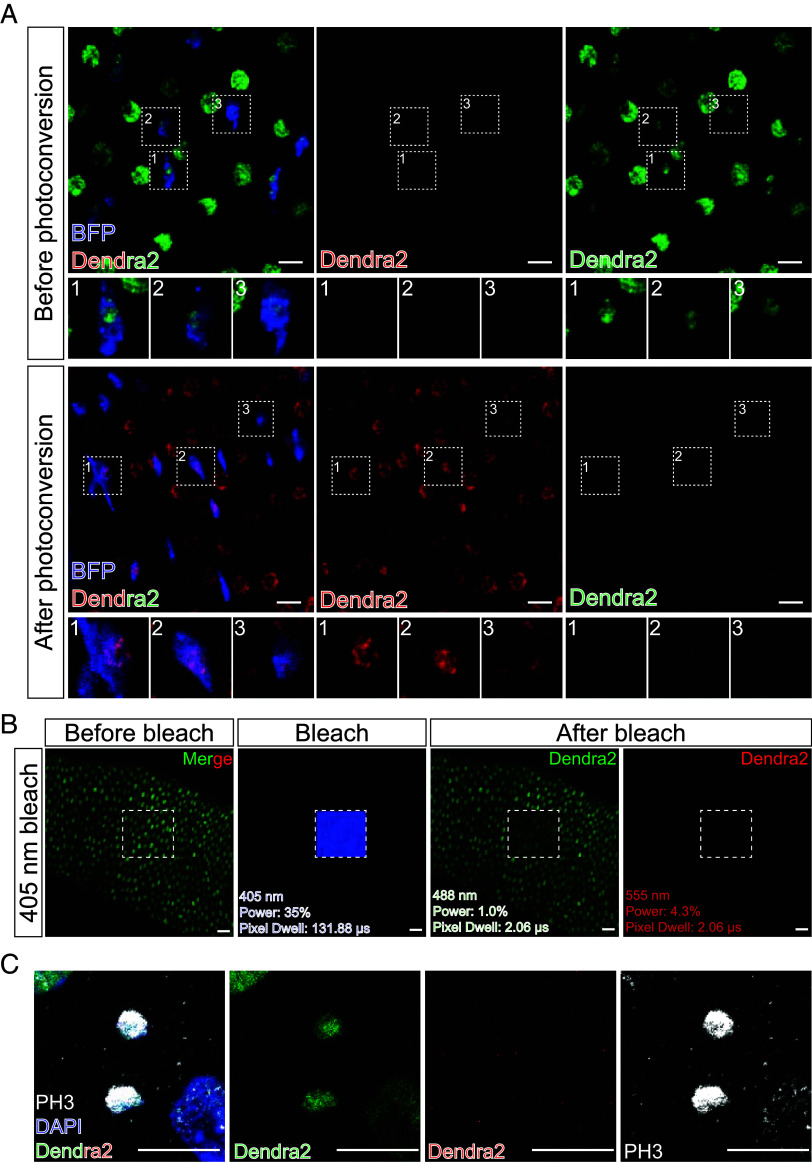
Technical controls for Histone-Dendra2 imaging and analysis. (*A*) Cell-to-cell variation in Dendra2 expression levels [corresponding to Li et al. (2)’s figure 1C]. White dashed boxes (numbered 1 to 3) in the “Before” and “After” images demarcate distinct esg^+^ cells with varying signal intensities. (*B*) The 405 nm laser is incapable of photoswitching fixed H3.1-Dendra2. Sequential images show the same region before bleaching (initial state), during bleaching with a high-power 405 nm laser (white square), and after bleaching under initial imaging conditions. (*C*) The PH3 staining does not bleed through to the red Dendra2 channel in nonphotoswitched samples. Using the imaging setting we used in the paper (2), the corresponding 555 nm channel shows no detectable signal. [Scale bar: 10 μm (*A* and *B*); 5 μm (*C*).]

Chen raised specific concerns regarding photoconversion efficiency and potential imaging artifacts, all of which we have addressed through dedicated control experiments detailed in our full response (3). Regarding photoconversion efficiency, the variation in postconversion red-to-green fluorescence ratios stems from inherent cell-to-cell differences in total H3-Dendra2 protein expression, not from incomplete conversion [Fig. 2*A* and (3)]. Crucially, following our in vivo photoconversion protocol, the green-Dendra2 signal is reduced to background levels in every analyzed esg^+^ cell, confirming highly efficient conversion (Fig. 2*A*). To rule out the possibility of accidental photoswitching during confocal imaging, we performed sequential high-power laser illumination tests on fixed samples. We demonstrated that neither the 405 nm nor the 488 nm laser lines on our microscope, even at elevated power, could generate the red fluorescence of photoconverted Dendra2; they could only cause photobleaching [Fig. 2*B* and (3)]. This confirms that our standard image acquisition settings do not artifactually create the “old” histone signal. Finally, regarding potential spectral bleed-through, we demonstrate that the PH3 signal detected in the far-red channel (Alexa Fluor 647) is absent in the red Dendra2 channel under our imaging conditions, thereby confirming the quantitative accuracy of our segregation measurements (Fig. 2*C*). Together, these controls validate the integrity of our photoconversion-based tracking method.

In summary, data obtained using endogenously regulated histones, backed by stringent technical validation, robustly demonstrate symmetric segregation of old and new H3.1 histones in each of the several asymmetric stem cell divisions assayed in our study.
